# Supplementary material for: Household perceptions, practices, and experiences with real-world alternating dual-pit latrines treated with storage and lime in rural Cambodia
Source: PLoS One. 2025 Oct 17;20(10):e0332118. doi: 10.1371/journal.pone.0332118 (PMC12533883; doi:10.1371/journal.pone.0332118)
Supplement: S1 File — (DOCX) [file pone.0332118.s001.docx]

## ADP Installation Manual and Technical Guidelines, Behavior Survey, and Collected Data

The ADP Installation Manual and Technical Guidelines; Behavior Survey developed for this study and administered using Taroworks; and data collected for this study are available at <https://osf.io/uwq82/>.
