# Supplementary material for: Household perceptions, practices, and experiences with real-world alternating dual-pit latrines treated with storage and lime in rural Cambodia
Source: PLoS One. 2025 Oct 17;20(10):e0332118. doi: 10.1371/journal.pone.0332118 (PMC12533883; doi:10.1371/journal.pone.0332118)
Supplement: S2 File — (DOCX) [file pone.0332118.s002.docx]

## Inclusion Criteria

All households included in the study met specific inclusion criteria, which were determined by asking each household, since the installation of their ADP:

- Has their pit filled or overflowed?
- Has their pit been pierced^[[1]](#footnote-2)^ or modified for fluid discharge?
- Has their pit been emptied, either partially or completely?
- Has their pit been switched from the new pit to the old pit?
- Is their toilet offset, and were their pits installed in parallel?

If a household member answered “yes” to any of the above questions, they were given the behavioral survey in person by research assistants, who are full-time trained staff of iDE’s monitoring and evaluation team.

1. Pit piercing is the practice of installing a hole or overflow pipe into the top ring of a pit to allow liquid FS to drain out of the top of a pit when it becomes nearly full. FS then drains into nearby bodies of water, onto open land, or into additional pits or tanks. Pit piercing is an unsafe practice because it contaminates the environment and typically exposes households and communities to harmful pathogens. [↑](#footnote-ref-2)
