# Supplementary material for: Household perceptions, practices, and experiences with real-world alternating dual-pit latrines treated with storage and lime in rural Cambodia
Source: PLoS One. 2025 Oct 17;20(10):e0332118. doi: 10.1371/journal.pone.0332118 (PMC12533883; doi:10.1371/journal.pone.0332118)
Supplement: S3 File — (DOCX) [file pone.0332118.s003.docx]

## Description of the Study Population

More households were located in Svay Rieng and Prey Veng provinces, and the fewest were located in Kandal province (Table S3.1).

Table S3.1. Number of Districts, Villages, and Households in Study Population, Disaggregated by Province

| Provinces | Districts | Villages | Households |
| --- | --- | --- | --- |
| Kampong Thom | 8 | 81 | 149 20% |
| Kandal | 7 | 40 | 94 12% |
| Prey Veng | 13 | 128 | 194 25% |
| Siem Reap | 12 | 91 | 140 18% |
| Svay Rieng | 8 | 112 | 188 25% |
| TOTAL | 40 | 452 | 765 |

Nearly two thirds of respondents (63%) were women, which likely occurred because men were more likely to be working outside of the household when surveying occurred (Table S3.2).

Table S3.2. Gender of Respondents in Study Population, Disaggregated by Province

| Province | % Respondents | |
| --- | --- | --- |
|  | Female | Male |
| Kampong Thom | 70% | 30% |
| Kandal | 59% | 41% |
| Prey Veng | 62% | 38% |
| Siem Reap | 65% | 35% |
| Svay Rieng | 58% | 42% |

Approximately two thirds of households (540) were located in an area identified as flood-prone, which indicates that a household is likely to experience at least one flood event within a year (Table S3.3). The high fraction of households living in flood-prone areas can be explained by the fact that the majority of communities in the five target provinces are located in close proximity to water bodies (e.g., lakes and rivers). This is common in rural Cambodia and indicates that climate vulnerability (i.e., having a higher likelihood of experiencing effects from climate events) is a mainstream issue for the majority of ADP customers.

Table S3.3. Flood Proneness of Households in Study Population, Disaggregated by Province

| Province | % Households that Are Considered Flood-prone |
| --- | --- |
| Kampong Thom | 85% |
| Kandal | 61% |
| Prey Veng | 68% |
| Siem Reap | 45% |
| Svay Rieng | 84% |

Only 6% of households in the study population were identified as poor (either IDPoor 1 or IDPoor 2; Table S3.4).

Table S3.4. Poverty in Study Population, Disaggregated by Province

| Province | % Households that Were Identified as Poor | |
| --- | --- | --- |
|  | IDPoor1 | IDPoor2 |
| Kampong Thom | 0% | 1% |
| Kandal | 0% | 3% |
| Prey Veng | 1% | 5% |
| Siem Reap | 2% | 3% |
| Svay Rieng | 3% | 4% |
| All households | 6% | |

Two thirds of households reported that at least one household member had completed secondary school (Table S3.5). One-in-five households reported at least one member with a university degree, and only one-in-ten households reported that primary school was the highest level of education received by any member.

Table S3.5. Education Achieved by At Least One Household Member in Study Population, Disaggregated by Province

| Province | % Households That Had At Least One Household Member that Achieved Education Level | | | | | |
| --- | --- | --- | --- | --- | --- | --- |
|  | No Formal Education | Primary | Secondary | University | Vocational Training |  |
| Kampong Thom | 1% | 15% | 65% | 19% | 0% |  |
| Kandal | 2% | 11% | 58% | 26% | 3% |  |
| Prey Veng | 0% | 15% | 61% | 24% | 0% |  |
| Siem Reap | 0% | 13% | 75% | 13% | 0% |  |
| Svay Rieng | 0% | 7% | 70% | 22% | 1% |  |
| All households | 0% | 13% | 66% | 21% | 1% |  |

The latrines of households in the study population were used by an average of 8.2 people daily. A majority of households did not share their latrine with anyone outside of their household (63%), and the average number of latrine users within these households was 6.2. The remaining 37% of households shared their latrine with household members and, on average, 5.3 individuals from outside of the household.

Nearly all households had at least one existing pit prior to ADP installation (99%), with only five households being unsure if they had had an existing pit prior to ADP installation. Two thirds of households had only one pit when their ADP was installed (70%). Of households that had at least two pits prior to ADP installation (30%), nearly all (99% or 222 households) had connected their old and new pits, while 88% had only connected a single pit.

Of households whose pits were connected in a series prior to ADP installation, most had an old pit with one, two or three concrete rings underground (94%), and two thirds of these households’ pits were equipped with three concrete rings (69%).
