# Supplementary figures and images for: Household perceptions, practices, and experiences with real-world alternating dual-pit latrines treated with storage and lime in rural Cambodia
Source: PLoS One. 2025 Oct 17;20(10):e0332118. doi: 10.1371/journal.pone.0332118 (PMC12533883; doi:10.1371/journal.pone.0332118)

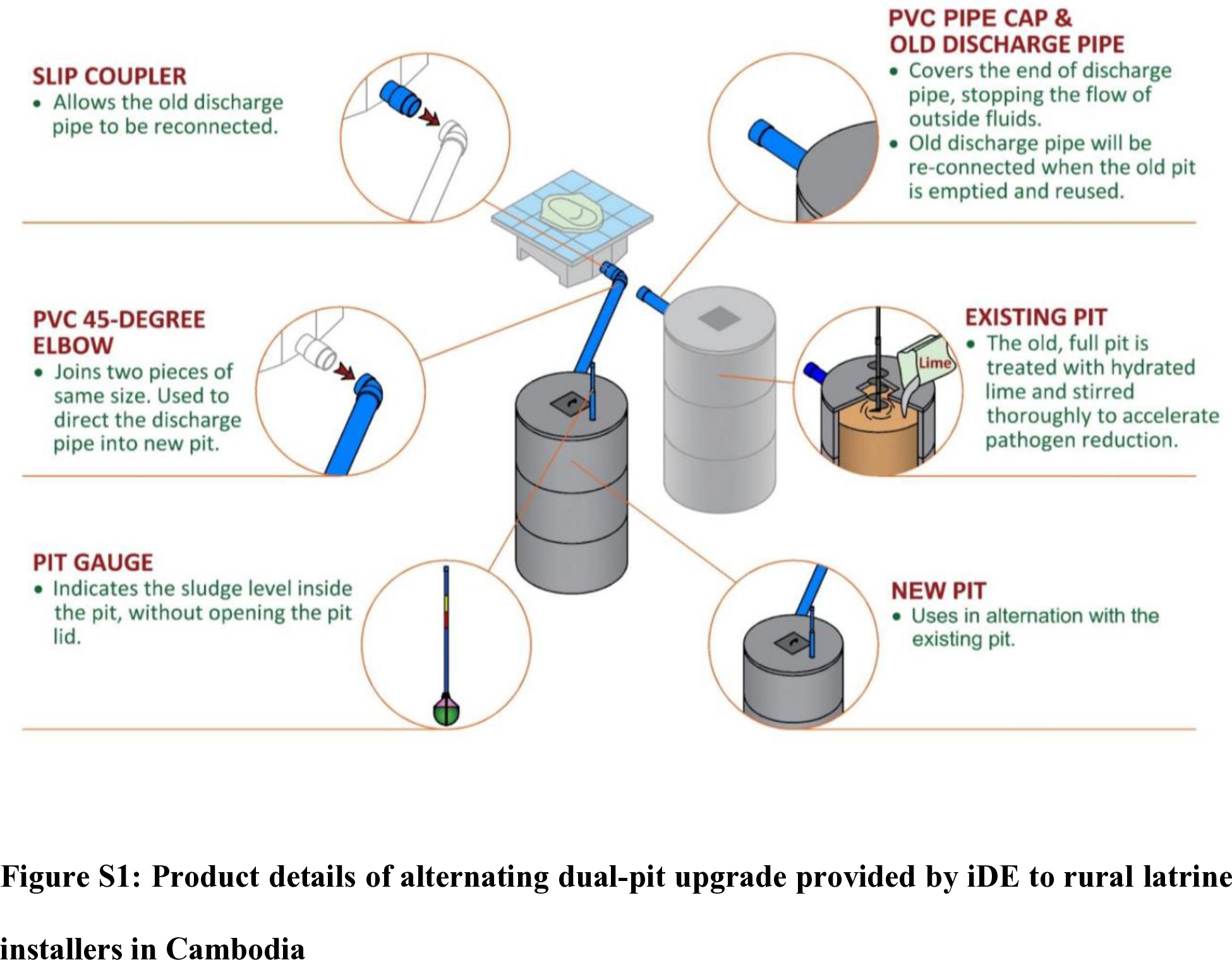

Supplement: S1 Fig — (TIF) [file pone.0332118.s004.tif]

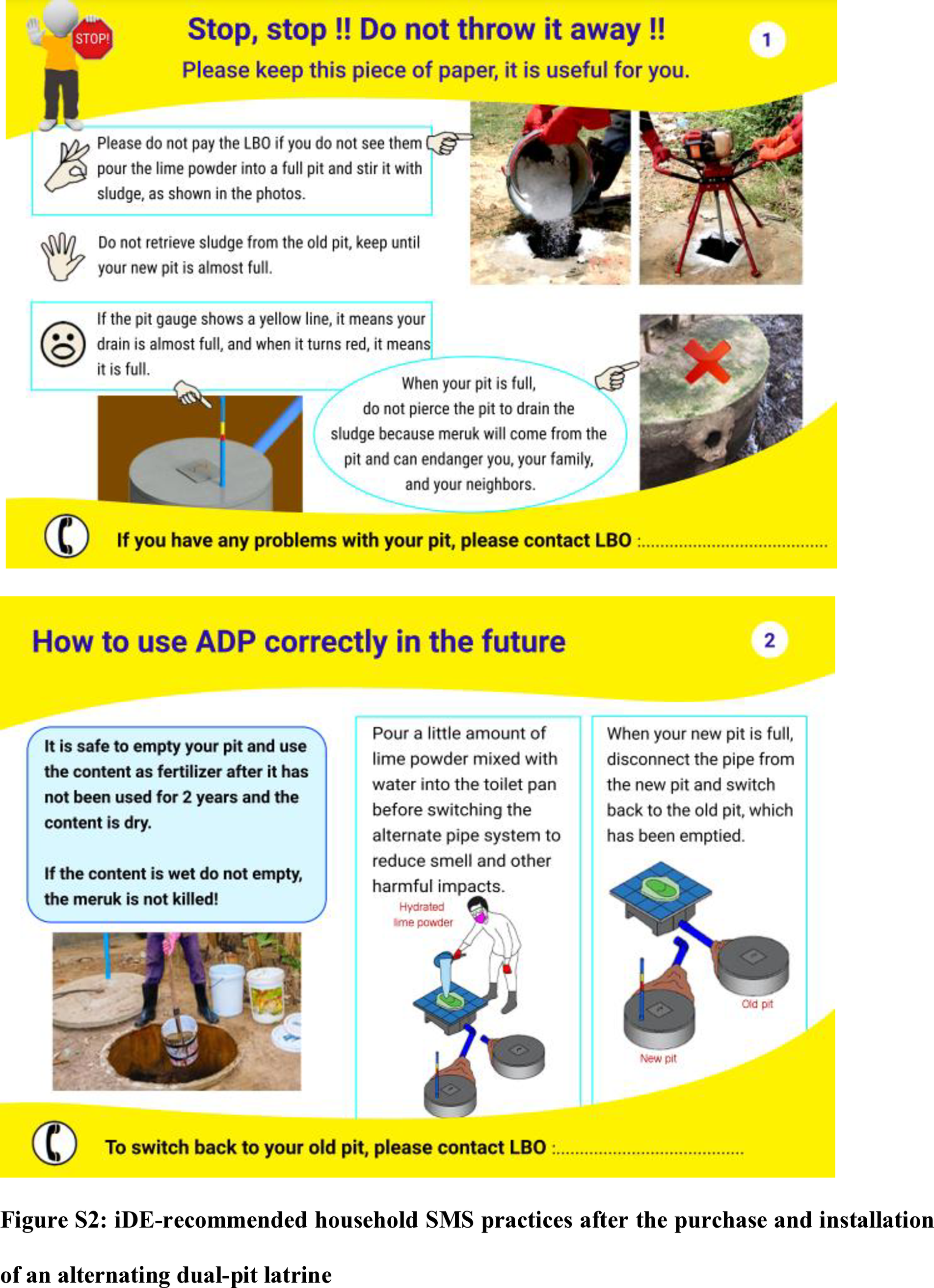

Supplement: S2 Fig — (TIF) [file pone.0332118.s005.tif]
