# Supplementary material for: Household perceptions, practices, and experiences with real-world alternating dual-pit latrines treated with storage and lime in rural Cambodia
Source: PLoS One. 2025 Oct 17;20(10):e0332118. doi: 10.1371/journal.pone.0332118 (PMC12533883; doi:10.1371/journal.pone.0332118)
Supplement: S1 Table — (DOCX) [file pone.0332118.s006.docx]

Table S1. Sample size calculations

| Province | Total Number of ADPs that Have Been in Use for ≥ 2 Years | ADPs that Have Likely Filled Since Installation | Calculated Household Sample Size to Achieve Desired Precision |
| --- | --- | --- | --- |
| Kampong Thom | 1,377 | 345 | 151 |
| Kandal | 582 | 146 | 95 |
| Prey Veng | 2,843 | 711 | 195 |
| Siem Reap | 1,103 | 276 | 136 |
| Svay Rieng | 2,505 | 627 | 188 |
| Total | 8,410 | 2,105 | 765 |
| Note: A pit fill rate of 25% was assumed across all provinces based on phone survey data. | | | |
