# Supplementary material for: Household perceptions, practices, and experiences with real-world alternating dual-pit latrines treated with storage and lime in rural Cambodia
Source: PLoS One. 2025 Oct 17;20(10):e0332118. doi: 10.1371/journal.pone.0332118 (PMC12533883; doi:10.1371/journal.pone.0332118)
Supplement: S2 Table — (DOCX) [file pone.0332118.s007.docx]

Table S2. Five ADP Indices with the survey questions used to construct them

| Index | Survey Question |
| --- | --- |
| Emptying  Practices | Did your household empty your old pit? |
|  | Who emptied your old pit? |
|  | What methods were used to empty your old pit? |
|  | Did your household pierce your pit (i.e., install an overflow hole)? |
|  | How many months was your old pit left disconnected from your toilet before it was emptied? |
|  | Where was the FS disposed of? |
|  | How was the FS disposed of? |
| Switching Practices | When your household switched pits, was your old pit emptied when your new pit filled? |
|  | When your household switched pits, who emptied your old pit? |
|  | When your household switched pits, how many months was your old pit left disconnected from your toilet before it was emptied? |
|  | Who switched the pits? |
|  | When your household switched pits, was your old pit disconnected from your toilet until your new pit filled? |
| Treatment  Practices | When your household switched pits, was the pit most recently connected to your toilet treated with lime? |
|  | When your household switched pits, who performed the lime treatment in the pit most recently connected to the toilet? |
|  | Was any product used to treat the FS in the pit most recently connected to the toilet? |
| Sanitation  Knowledge | Can FS affect your and your family’s health? |
|  | Is disposing FS into a body of water unsafe? |
|  | Is disposing FS onto a field unsafe? |
|  | Is the water that comes out of a pit above ground unsafe? |
| Sanitation  Attitudes | Is killing pathogens in FS important to your household? |
|  | Is safety important to your household when considering where to dispose of FS? |
|  | Is a household member emptying a pit containing untreated FS unsafe? |
