# Supplementary material for: Household perceptions, practices, and experiences with real-world alternating dual-pit latrines treated with storage and lime in rural Cambodia
Source: PLoS One. 2025 Oct 17;20(10):e0332118. doi: 10.1371/journal.pone.0332118 (PMC12533883; doi:10.1371/journal.pone.0332118)
Supplement: S3 Table — (DOCX) [file pone.0332118.s008.docx]

Table S3. Five ADP Indices with associated questions, possible responses, scores, and score rationales

| Index | Question | Possible Responses | Scores | Score Rationale |
| --- | --- | --- | --- | --- |
| Emptying Practices | Did the household empty their old pit? | Yes  No | 0  2 | Because all households in this study reported at least one pit fill, emptying their pit when it filled is the first step in selecting a safe FSM practice. |
|  | Who emptied the old pit? | Household member  Friend or neighbor  Service provider  Other | 0  0  2  0 | Being aware of safer FSM practices (e.g., hiring a trained service provider) is a key component of performing a safer FSM practice. Less safe emptying practices that are typically performed in rural contexts include a household emptying their own pit (e.g., self-empty). |
|  | What methods were used to empty the old pit? | Manually  Mechanically Vacuum truck Buried old pit  Don't know | 0  1  2  0  0 | Pit emptying should be performed using specific equipment with proper safety techniques to prevent the release of untreated FS into the environment. Vacuum trucks typically provide the safest emptying of FS. Emptying mechanically (e.g., with a pump and piping) is typically less safe than a vacuum truck due to leaks and exposure to sludge, and emptying manually (e.g., with a shovel) is least safe. |
|  | Did the household pierce their pit? | Yes  No or Don't know | 0  2 | Piercing a pit is a dangerous practice and releases untreated FS into the environment. |
|  | How many months was the old pit left disconnected from the toilet before it was emptied? | < 1 year  1-1.5 years  >2 years  Don't know | 0  1  2  0 | Believing that FS can be emptied safely in less than 1 year is dangerous. Understanding that FS becomes safer to empty after 2 years indicates that a household understands how ADPs work. |
|  | Where was the FS disposed of?’ | <10-sec walk…  <1-min walk…  >1-min walk…  …from pit, house, or water point  Don't know | 0  1  2  0 | Households tend to select locations within their communities or nearby their houses for FS disposal, which can expose people to the pathogens in untreated FS. |
|  | How was the waste disposed of? | Buried  Into body of water  Onto field as waste  Onto field as fertilizer  Into treatment plant  Don't know | 2  0  0  1  2  0 | Safer FSM practices include disposal of FS into a landfill or reused after treatment, typically as fertilizer or fuel. |
| Switching Practices | When switched pits, was the old pit emptied when the new pit filled? | Yes  No | 2  0 | Households should have emptied their disconnected pit before the new pit filled. |
|  | When switched pits, who emptied the old pit? | Household member  Friend or neighbor  Service provider  Don't know | 0  0  2  0 | Households should have had their disconnect pit emptied by a trained service provider. |
|  | When switched pits, how many months was the old pit left disconnected from the toilet before it was emptied? | < 1 year  1-1.5 years  >2 years  Don't know | 0  1  2  0 | The household should have left the old pit disconnected for a sufficient amount of time (at least two years) before emptying. |
|  | Who switched the pits? | Household member  Service provider | 0  2 | The household should have hired a trained service provider to switch their pits. |
|  | When switched pits, was the old pit disconnected from the toilet until the new pit filled? | Yes  No | 2  0 | The household should have disconnected their old pit from the toilet until the new pit filled. |
| Treatment Practices | When switched pits, was the pit most recently connected to the toilet treated with lime? | Yes  No or Don’t know | 2  0 | The full pit should have been treated with lime after the connection was switched. |
|  | When switched pits, who performed the lime treatment in the pit most recently connected to the toilet? | Household member  Service provider | 0  2 | Lime treatment should have been performed by a qualified service provider. |
|  | Was any product used to treat the FS in the pit most recently connected to the toilet? | Yes (2)  No or Don't know | 2  0 | Adding a product to treat/kill meruk (substances that cause disease or bad smells) in FS is important. |
| Sanitation Knowledge | Know that FSM affects health | No  Somewhat  Yes | 0  1  2 | Understanding that how pits are managed affects public health indicates more knowledge about safer FSM practices. |
|  | Know that disposing FS into a body of water is unsafe | No  Somewhat  Yes | 0  1  2 | Understanding that putting FS in bodies of water is unsafe indicates more knowledge about safer FSM practices. |
|  | Know that disposing FS onto a field is unsafe | No  Somewhat  Yes | 0  1  2 | Understanding that putting FS onto fields of water is unsafe indicates more knowledge about safer FSM practices. |
|  | Know that the water that comes out of a pit above ground is unsafe | No  Somewhat  Yes | 0  1  2 | Understanding that water from pits is unsafe indicates more knowledge about safer FSM practices. |
| Sanitation Attitudes | Feels that killing pathogens in FS is important | No  Somewhat  Yes | 0  1  2 | Feeling that killing meruk in FS (i.e., treatment) is important indicates more positive attitudes towards safer FSM practices. |
|  | Feels that safety is a top consideration when considering where to dispose of FS | No  Somewhat  Yes | 0  1  2 | Feeling that choosing a safe location to dispose FS is important indicates more positive attitudes towards safer FSM practices. |
|  | Feels that a household member emptying a pit is unsafe | No  Somewhat  Yes | 0  1  2 | Feeling that it is not right for the HH member to empty the pit because they are not a professional service provider indicates more positive attitudes towards safer FSM practices. |
