# Supplementary material for: Household perceptions, practices, and experiences with real-world alternating dual-pit latrines treated with storage and lime in rural Cambodia
Source: PLoS One. 2025 Oct 17;20(10):e0332118. doi: 10.1371/journal.pone.0332118 (PMC12533883; doi:10.1371/journal.pone.0332118)
Supplement: S4 Table — (DOCX) [file pone.0332118.s009.docx]

Table S4. Linear Regression Results of the Emptying Practices Index

| Variable^1^ | Emptying Practices Index | |
| --- | --- | --- |
|  | Coefficient with  Standard Error and  95% Confidence Interval | *p*-value |
| Province | | |
| Kampong Thom | - | - |
| Kandal | 0.04 (0.03) -0.02 to 0.10 | 0.5 |
| Prey Veng | -0.20*** (0.03) -0.26 to -0.14 | 0.000 |
| Siem Reap | 0.13** (0.04) 0.05 to 0.21 | 0.02 |
| Svay Rieng | -0.40*** (0.05) -0.50 to -0.30 | 0.000 |
| Flood proneness | | |
| Non-flood prone | - | - |
| Flood-prone | 0.0 (0.4) -0.8 to 0.8 | 0.9 |
| Poverty level (IDPoor status) | | |
| Non-IDPoor | - | - |
| IDPoor 1 | 0.3 (0.1) 0.1 to 0.5 | 0.1 |
| IDPoor 2 | -0.1 (0.2) -0.5 to 0.3 | 0.4 |
| Unknown | 0.15* (0.05) 0.05 to 0.25 | 0.07 |
| Education |  |  |
| No formal education | - | - |
| Primary schooling | -0.20** (0.04) -0.28 to -0.12 | 0.02 |
| Secondary schooling | -0.15** (0.03) -0.21 to -0.09 | 0.04 |
| University graduate | -0.1 (0.2) -0.5 to 0.3 | 0.3 |
| Vocational training | -0.2 (0.3) -0.8 to 0.4 | 0.5 |
| # times pit overflowed since ADP installed | | |
| Never | - | - |
| 1-3 times | -0.58** (0.06) -0.70 to -0.46 | 0.01 |
| 4-10 times | -0.61** (0.07) -0.75 to -0.47 | 0.01 |
| More than 10 times | -0.66*** (0.07) -0.80 to -0.52 | 0.007 |
| Constant | 1.6*** (0.1) 1.4 to 1.8 | 0.000 |
| Observations | 700 | |
| Adjusted R-Squared | 0.12 | |

1: All coefficients of categorical variables are in reference to the first response indicated (e.g., “Non-IDPoor” and “No formal education”). Thus, all coefficients describe the difference between a given response and the reference response.

* p<0.1; ** p<0.05; *** p<0.01
