# Supplementary material for: Household perceptions, practices, and experiences with real-world alternating dual-pit latrines treated with storage and lime in rural Cambodia
Source: PLoS One. 2025 Oct 17;20(10):e0332118. doi: 10.1371/journal.pone.0332118 (PMC12533883; doi:10.1371/journal.pone.0332118)
Supplement: S5 Table — (DOCX) [file pone.0332118.s010.docx]

Table S5. Linear Regression Results of the Treatment Practices Index

| Variable^1^ | Treatment Practices Index | |
| --- | --- | --- |
|  | Coefficient with  Standard Error and  95% Confidence Interval | p-value |
| Province | | |
| Kampong Thom | - | - |
| Kandal | 0.1 (0.2) -0.3 to 0.5 | 0.6 |
| Prey Veng | 0.1 (0.2) -0.3 to 0.5 | 0.4 |
| Siem Reap | 0.0 (0.3) -0.6 to -0.6 | 0.8 |
| Svay Rieng | 0.2 (0.1) 0.0 to 0.2 | 0.2 |
| Flood proneness | | |
| Non-flood prone | - | - |
| Flood-prone | -0.19** (0.03) -0.25 to -0.13 | 0.02 |
| Poverty level (IDPoor status) | | |
| Non-IDPoor | - | - |
| IDPoor 1 | 1.27*** (0.07) 1.13 to 1.41 | 0.000 |
| IDPoor 2 | -0.2 (0.2) -0.6 to 0.2 | 0.3 |
| Unknown | -0.18* (0.05) -0.28 to -0.08 | 0.06 |
| Education |  |  |
| No formal education | - | - |
| Primary schooling^2^ | - | - |
| Secondary schooling | 0.1 (0.1) -0.1 to 0.3 | 0.2 |
| University graduate | 0.0 (0.3) -0.6 to 0.6 | 0.9 |
| Vocational training | -0.2 (0.1) -0.4 to 0.0 | 0.2 |
| # times pit overflowed since ADP installed | | |
| Never | - | - |
| 1-3 times | 0.34*** (0.04) 0.26 to 0.42 | 0.000 |
| 4-10 times | 0.2 (0.1) 0.0 to 0.4 | 0.2 |
| More than 10 times | 0.57*** (0.07) 0.42 to 0.71 | 0.008 |
| Constant | -0.1 (0.2) -0.5 to 0.3 | 0.5 |
| Observations | 311 | |
| Adjusted R-Squared | 0.07 | |
|  |  | |

1: All coefficients of categorical variables are in reference to the first response indicated (e.g., “Non-IDPoor” and “No formal education”). Thus, all coefficients describe the difference between a given response and the reference response.

2: Too few households reported this response; thus, this category was removed from this model.

* p<0.1; ** p<0.05; *** p<0.01
