# Supplementary material for: Household perceptions, practices, and experiences with real-world alternating dual-pit latrines treated with storage and lime in rural Cambodia
Source: PLoS One. 2025 Oct 17;20(10):e0332118. doi: 10.1371/journal.pone.0332118 (PMC12533883; doi:10.1371/journal.pone.0332118)
Supplement: S6 Table — (DOCX) [file pone.0332118.s011.docx]

Table S6. Linear Regression Results of the Switching Practices Index

| Variable^1^ | Switching Practices Index | |
| --- | --- | --- |
|  | Coefficient with  Standard Error and  95% Confidence Interval | *p*-value |
| Province | | |
| Kampong Thom | - | - |
| Kandal | 0.1 (0.2) -0.3 to 0.5 | 0.4 |
| Prey Veng | 0.0 (0.2) -0.4 to 0.4 | 0.5 |
| Siem Reap | 0.0 (0.6) -1.2 to 1.2 | 1.0 |
| Svay Rieng | 0.0 (0.5) -1.0 to 1.0 | 0.9 |
| Flood proneness | | |
| Non-flood prone | - | - |
| Flood-prone | 0.0 (0.4) -0.8 to 0.8 | 0.4 |
| Poverty level (IDPoor status) | | |
| Non-IDPoor | - | - |
| IDPoor 1 | -0.45* (0.07) 0.31 to 0.59 | 0.08 |
| IDPoor 2 | -0.1 (0.4) -0.9 to 0.7 | 0.7 |
| Unknown | 0.2 (0.3) -0.4 to 0.8 | 0.4 |
| Education |  |  |
| No formal education | - | - |
| Primary schooling^2^ | - | - |
| Secondary schooling | 0.0 (0.5) -1.0 to 1.0 | 0.8 |
| University graduate | 0.0 (0.6) -1.2 to 1.2 | 1.0 |
| Vocational training | -0.14* (0.04) -0.22 to -0.06 | 0.08 |
| # times pit overflowed since ADP installed | | |
| Never | - | - |
| 1-3 times | -0.1 (0.3) -0.7 to 0.5 | 0.4 |
| 4-10 times | 0.0 (0.6) -1.2 to 1.2 | 0.8 |
| More than 10 times | 0.0 (0.7) -1.4 to 1.4 | 0.9 |
| Constant | 0.94*** (0.12) 0.70 to 1.18 | 0.000 |
| Observations | 211 | |
| Adjusted R-Squared | -0.01 | |

1: All coefficients of categorical variables are in reference to the first response indicated (e.g., “Non-IDPoor” and “No formal education”). Thus, all coefficients describe the difference between a given response and the reference response.

2: Too few households reported this response and thus, this category was removed from this model.

* p<0.1; ** p<0.05; *** p<0.01
