# Supplementary material for: Household perceptions, practices, and experiences with real-world alternating dual-pit latrines treated with storage and lime in rural Cambodia
Source: PLoS One. 2025 Oct 17;20(10):e0332118. doi: 10.1371/journal.pone.0332118 (PMC12533883; doi:10.1371/journal.pone.0332118)
Supplement: S7 Table — (DOCX) [file pone.0332118.s012.docx]

Table S7. Linear Regression Results of the Sanitation Knowledge Index

| Variable^1^ | Sanitation Knowledge Index | |
| --- | --- | --- |
|  | Coefficient with  Standard Error and  95% Confidence Interval | *p*-value |
| Province | | |
| Kampong Thom | - | - |
| Kandal | -0.11** (0.02) -0.15 to -0.07 | 0.04 |
| Prey Veng | 0.0 (0.3) -0.6 to 0.6 | 0.4 |
| Siem Reap | 0.12** (0.02) 0.08 to 0.16 | 0.02 |
| Svay Rieng | 0.0 (0.5) -1.0 to 1.0 | 0.9 |
| Flood proneness | | |
| Non-flood prone | - | - |
| Flood-prone | 0.1 (0.2) -0.3 to 0.5 | 0.2 |
| Poverty level (IDPoor status) | | |
| Non-IDPoor | - | - |
| IDPoor 1 | 0.24*** (0.05) 0.14 to 0.34 | 0.002 |
| IDPoor 2 | -0.1 (0.3) -0.7 to 0.5 | 0.5 |
| Unknown | -0.1 (0.4) -0.9 to 0.7 | 0.6 |
| Education |  |  |
| No formal education | - | - |
| Primary schooling | -0.21* (0.06) -0.33 to -0.09 | 0.08 |
| Secondary schooling | -0.2 (0.1) -0.4 to 0.0 | 0.1 |
| University graduate | -0.2 (0.2) -0.6 to 0.2 | 0.2 |
| Vocational training | -0.42*** (0.03) -0.48 to -0.36 | 0.005 |
| # times pit overflowed since ADP installed | | |
| Never | - | - |
| 1-3 times | -0.16* (0.05) -0.26 to -0.06 | 0.09 |
| 4-10 times | -0.1 (0.2) -0.5 to 0.3 | 0.5 |
| More than 10 times | -0.20* (0.05) -0.30 to -0.10 | 0.08 |
| Constant | 1.46*** (0.17) 1.13 to 1.80 | 0.000 |
| Observations | 700 | |
| Adjusted R-Squared | 0.03 | |

1: All coefficients of categorical variables are in reference to the first response indicated (e.g., “Non-IDPoor” and “No formal education”). Thus, all coefficients describe the difference between a given response and the reference response.

* p<0.1; ** p<0.05; *** p<0.01
