# Supplementary material for: Household perceptions, practices, and experiences with real-world alternating dual-pit latrines treated with storage and lime in rural Cambodia
Source: PLoS One. 2025 Oct 17;20(10):e0332118. doi: 10.1371/journal.pone.0332118 (PMC12533883; doi:10.1371/journal.pone.0332118)
Supplement: S8 Table — (DOCX) [file pone.0332118.s013.docx]

Table S8. Linear Regression Results of the Sanitation Attitudes Index

| Variable^1^ | Sanitation Attitudes Index | |
| --- | --- | --- |
|  | Coefficient with  Standard Error and  95% Confidence Interval | *p*-value |
| Province | | |
| Kampong Thom | - | - |
| Kandal | -0.09** (0.03) -0.15 to -0.03 | 0.04 |
| Prey Veng | -0.19*** (0.04) -0.27 to -0.11 | 0.000 |
| Siem Reap | -0.09** (0.03) -0.15 to -0.03 | 0.02 |
| Svay Rieng | -0.14*** (0.03) -0.20 to -0.08 | 0.001 |
| Flood proneness | | |
| Non-flood prone | - | - |
| Flood-prone | -0.09*** (0.03) -0.15 to -0.03 | 0.001 |
| Poverty level (IDPoor status) | | |
| Non-IDPoor | - | - |
| IDPoor 1 | 0.1 (0.2) -0.3 to 0.5 | 0.4 |
| IDPoor 2 | 0.1 (0.2) -0.3 to 0.5 | 0.3 |
| Unknown | -0.1 (0.2) -0.5 to 0.3 | 0.3 |
| Education |  |  |
| No formal education | - | - |
| Primary schooling | 0.2 (0.3) -0.4 to 0.8 | 0.4 |
| Secondary schooling | 0.2 (0.2) -0.2 to 0.6 | 0.3 |
| University graduate | 0.3 (0.2) -0.1 to 0.7 | 0.3 |
| Vocational training | 0.3 (0.2) -0.1 to 0.7 | 0.3 |
| # times pit overflowed since ADP installed | | |
| Never | - | - |
| 1-3 times | 0.0 (0.4) -0.8 to 0.8 | 0.8 |
| 4-10 times | 0.0 (0.4) -0.8 to 0.8 | 0.9 |
| More than 10 times | 0.1 (0.3) -0.5 to 0.7 | 0.6 |
| Constant | 1.73*** (0.19) 1.36 to 2.10 | 0.000 |
| Observations | 700 | |
| Adjusted R-Squared | 0.03 | |

1: All coefficients of categorical variables are in reference to the first response indicated (e.g., “Non-IDPoor” and “No formal education”). Thus, all coefficients describe the difference between a given response and the reference response.

* p<0.1; ** p<0.05; *** p<0.01
