# Supplementary material for: Household perceptions, practices, and experiences with real-world alternating dual-pit latrines treated with storage and lime in rural Cambodia
Source: PLoS One. 2025 Oct 17;20(10):e0332118. doi: 10.1371/journal.pone.0332118 (PMC12533883; doi:10.1371/journal.pone.0332118)
Supplement: Attachment — Submitted filename: Response to Reviewers.docx [file pone.0332118.s015.docx]

Response to Reviewers

PONE-D-24-32665

Household Perceptions, Practices, and Experiences with Real-world Alternating Dual-Pit Latrines Treated with Storage and Lime in Rural Cambodia

James Harper, PE, PhD and co-authors

May 9, 2025

Thank you for your comments on this article. We have addressed and responded to all of your comments and believe that it is now ready for publishing with PLOS ONE. Please see below for details.

# EDITOR

## Comment 1

Thank you for submitting your manuscript to PLOS ONE. After careful consideration, we feel that it has merit but does not fully meet PLOS ONE’s publication criteria as it currently stands. Therefore, we invite you to submit a revised version of the manuscript that addresses the points raised during the review process.

### Response

Thank you for the opportunity to revise this article. We have been careful to sufficiently address each reviewer comment listed below and hope that the changes we’ve made below are sufficient to address your and the reviewers’ concerns.

## Comment 2

Please include the following items when submitting your revised manuscript:

- A rebuttal letter that responds to each point raised by the academic editor and reviewer(s). You should upload this letter as a separate file labeled 'Response to Reviewers'.
- A marked-up copy of your manuscript that highlights changes made to the original version. You should upload this as a separate file labeled 'Revised Manuscript with Track Changes'.
- An unmarked version of your revised paper without tracked changes. You should upload this as a separate file labeled 'Manuscript'.
- If you would like to make changes to your financial disclosure, please include your updated statement in your cover letter. Guidelines for resubmitting your figure files are available below the reviewer comments at the end of this letter.

### Response

Thank you. We have adhered to these protocols in this resubmission.

## Comment 3

Please ensure that your manuscript meets PLOS ONE's style requirements, including those for file naming. The PLOS ONE style templates can be found at

<https://journals.plos.org/plosone/s/file?id=wjVg/PLOSOne_formatting_sample_main_body.pdf> and

<https://journals.plos.org/plosone/s/file?id=ba62/PLOSOne_formatting_sample_title_authors_affiliations.pdf>

### Response

Thank you. We have updated formatting throughout the document, including changing double-space text and changing citation formatting, per the PLOS ONE style templates.

## Comment 4

Thank you for stating the following financial disclosure: Grant funding provided by Australian Aid, Water for Women, USAID, and The Stone Family Foundation. Please state what role the funders took in the study. If the funders had no role, please state: ""The funders had no role in study design, data collection and analysis, decision to publish, or preparation of the manuscript."" If this statement is not correct you must amend it as needed. Please include this amended Role of Funder statement in your cover letter; we will change the online submission form on your behalf.

### Response

Thank you for this comment. Our financial disclosure has been updated to include the recommended sentence.

## Comment 5

Your ethics statement should only appear in the Methods section of your manuscript. If your ethics statement is written in any section besides the Methods, please delete it from any other section.

### Response

Thank you. This change has been made, and our ethics statement has been updated to describe our use of oral informed consent.

## Comment 6

We notice that your supplementary figures and tables are included in the manuscript file. Please remove them and upload them with the file type 'Supporting Information'. Please ensure that each Supporting Information file has a legend listed in the manuscript after the references list.

### Response

Thank you. We have updated the manuscript as described here and by the PLOS ONE style templates and requirements described in Comment 3.

## Comment 7

While revising your submission, please upload your figure files to the Preflight Analysis and Conversion Engine (PACE) digital diagnostic tool, https://pacev2.apexcovantage.com/. PACE helps ensure that figures meet PLOS requirements. To use PACE, you must first register as a user. Registration is free. Then, login and navigate to the UPLOAD tab, where you will find detailed instructions on how to use the tool. If you encounter any issues or have any questions when using PACE, please email PLOS at figures@plos.org. Please note that Supporting Information files do not need this step.

### Response

Thank you. We do not have any figures in the manuscript; there are only figures in the Supporting Information. So, this comment does not apply.

# REVIEWER 1

## Comment 1

The manuscript has been scientifically written evident with the data provided and the conclusion has been drawn after relevant statistical inference has been employed and justified. The statistical inference employed in this study answers the objective of the study assisted by the rigorous data collection techniques used in this study which was attached too. The layout and presentation of the study show a thorough cohesion throughout and is written in a manner that the reader can comprehend. With that said I recommend this submission for publication

### Response

Thank you for your kind comments. We hope that the changes we’ve made below are sufficient to address your and the reviewers’ concerns.

# REVIEWER 2

## Comment 1

You answered “No” to “Is the manuscript technically sound, and do the data support the conclusions? The manuscript must describe a technically sound piece of scientific research with data that supports the conclusions. Experiments must have been conducted rigorously, with appropriate controls, replication, and sample sizes. The conclusions must be drawn appropriately based on the data presented.”

### Response

Thank you for this feedback. We hope that the changes we’ve made below are sufficient to address your and the reviewers’ concerns.

## Comment 2

You answered “No” to “Has the statistical analysis been performed appropriately and rigorously?”

### Response

Thank you for this feedback. We hope that the changes we’ve made below are sufficient to address your and the reviewers’ concerns.

## Comment 3

This paper summarizes data collected during surveys of people using alternating dual-pit latrines (ADPs) across 5 rural provinces in Cambodia, a previously understudied safely managed sanitation system. Surveys collected information on sanitation management practices as well as knowledge and attitudes about ADP use. The authors used descriptive summaries of the data to assess the population’s sanitation practices and knowledge and developed indices summarizing various aspects of sanitation use to assess factors influencing sanitation practices, knowledge, and values. The authors conclude that the study population generally has poor sanitation knowledge and that reported ADP management consistently did not follow recommended practices. With results from the regression analyses, the authors further conclude that province, flood proneness, education, and other household factors impact people’s sanitation practices. My key concerns with this paper are that 1) there are issues with the quality and presentation of the methods and results sections, and 2) the conclusions do not align with the results presented in the paper. Specific concerns are outlined below.

### Response

Thank you for this feedback. We hope that the changes that we have made to the article are sufficient to address your and the other reviewers’ concerns.

## Comment 4

Methods: There was no description of the study population or survey participants, including the age, gender, etc. A summary table outlining the demographics of the population that responded to the survey would have been helpful. In particular, it would be helpful to describe any key differences between provinces.

### Response

Thank you for this feedback. We have added this information into the Supporting Information S3 and referenced this information in the main text in the Methods section’s “Study Design, Sampling Frame, Inclusion Criteria, and Sample Size” subsection.

## Comment 5

Methods: It is unclear why a “6% precision” rate was chosen to determine the sample size. Please provide a rationale, and ideally a citation to back up this choice.

### Response

Thank you for this feedback. In the Methods section, we have clarified why the study aimed to achieve the standard 5% precision but only achieved a 6% precision.

## Comment 6

Methods: In the way the authors developed the indices, all variables within each index are given the same weight, which may not be appropriate. Please provide a rationale for this decision and why a PCA or other type of factor analysis was not used to develop the indices.

### Response

Thank you for this feedback. We have added text in the Methods’ “Data Analysis” subsection to describe our choice to not weight the responses to certain survey questions to create each index. Also, to address the other aspect of your response, we initially performed an exploratory factor analysis and principle components analysis but ultimately were unable to explain the results clearly because the questions included in each factor were not easily relatable based on the literature and our experience in the local context of rural Cambodia. For example, explaining why one emptying practice would be included under one factor while another would not was unsupportable. We attributed the difficulty in explaining the EFA/PCA results to the complexities involved with explaining human decision-making, which is well documented in the literature. Thus, based on existing literature and experience in the local context, we opted to *a priori* specify which topics contributed to each index, which we believe provides an evidence-based and clear approach to describing relevant topics related to household perceptions, practices, and experiences with ADPs in rural Cambodia. This practice is also well documented in the literature and thereby supportable.

## Comment 7

Methods: The definition of “flood-prone” was not provided, and conclusions from results using that variable were often conflated to discussing climate change as a whole. In the methods section, the author notes including “climate vulnerability” in the model, but it is unclear if that is referring to flooding risk or if other climate considerations were assessed. The author should clearly define what makes a household flood-prone vs. not flood-prone and describe why flood-risk was used as an indicator of climate vulnerability beyond other measures (i.e. risk of drought, water availability, etc.).

### Response

Thank you for this feedback. We have modified the text to define “flood-proneness” in the Introduction and have modified various other locations in the text to use this term instead of “climate vulnerability” to improve clarity. We also added text to the Introduction to describe why we focused on flood-proneness in this study and did not consider other climate hazards like drought.

## Comment 8

Methods: It was unclear why the independent variables used in the regression model were chosen over others that were included in the survey. A theory of change diagram or other description would be helpful to understand why certain variables were included and not some of the other demographic factors listed in the survey. The independent variables seem at high risk of issues with multicollinearity (i.e. poverty and education) and overadjustment. Please provide a clear rationale for model development and variable selection. This could include correlation matrices to illustrate how the variables relate to one another in this specific sample.

### Response

Thank you for this feedback. We have added an explanation of how each survey question that was included in the calculation of a specific index was selected for inclusion and why others were not. We also added a discussion of collinearity and index development; for example, the factors you mention – poverty and education – were not included in any index (see Table S3) to mitigate colinearity between indices. However, these factors were included in the regressions because they have been shown to be associated with household sanitation practices, as shown in the literature cited in the original and new text in the Methods section. The different levels of poverty and education measured in this study did not show strong correlations with indicies, and only a few colinearities are noted in the regression results (e.g., poverty and vocational training in the Switching Practices index in Table S6). In most of the regression results, colinearities between poverty, education, and other demographics were not found (e.g., all other four indices in Tables S4, S5, S7, and S8).

## Comment 9

Conclusions: All conclusions about the regression model were drawn only based on p-values and no confidence intervals or estimates of variability were provided. This is not conventional and makes interpretation difficult. See, for example https://academic.oup.com/aje/article/186/6/627/4091005 and https://jamanetwork.com/journals/jama/fullarticle/2503156.

### Response

Thank you for this feedback. We apologize for this oversight and have modified Tables S4-8 to include standard errors and 95% confidence intervals. The results discussed in the text were based on a thorough evaluation of the regression models’ coefficients, standard errors, confidence intervals, and *p*-values; thus, the results in the text have not been modified.

## Comment 10

Conclusions: Conclusions about the regression models do not accurately describe model estimates in terms of their reference category. For example, the author notes that "households in Prey Veng and Svay Rieng followed recommended emptying practices less frequently and had worse sanitation attitudes compared to other provinces". This statement suggests that models are comparing individual provinces to all other provinces, but the models include province on a categorical scale with Kampong Thom as the reference category. With this approach, it is not appropriate to draw conclusions about one province compared to all others, where regression coefficients are only comparing each province to the reference.

### Response

Thank you for this feedback. We have modified the text in the Results section to describe these results more clearly and as you explained.

## Comment 11

Conclusions: Some conclusions made by the authors don’t seem to align with the presented data. For example, the author concludes that “households valued the advantages of ADPs”, when only a small percentage (at most 23%) reported valuing specific aspects of ADPs.

### Response

Thank you for this feedback. The original sentence at the beginning of the Results section was unclear, which led to your understandable misunderstanding of the results. The original sentence did not explicitly state how many households appreciated the advantages of ADPs but rather only stated a list of what households liked about ADPs, of which there were >10 less frequently reported and “Other” responses that we did not report in the article. To clarify these results, this sentence has been updated to show that 78% of households appreciated their ADP. Thus, we did not change the text in the Conclusion about how households appreciate the advantages of ADPs.

## Comment 12

Conclusions: The author should be careful not to conflate the issues of knowledge, attitudes, and practices in conclusions. For example, the author states that “households with poor sanitation knowledge did not highly value SMS and are therefore more likely to not empty their pit before it overflows”. Sanitation knowledge is not necessarily correlated with people’s values. It's very possible for people to not know a lot about sanitation and still value it.

### Response

Thank you for this feedback. We have reviewed and appropriately modified the sentence you noted. We have also reviewed the entire Conclusion section for similar conflations and made minor changes to improve clarity.

## Comment 13

Conclusions: The conclusion section makes strong recommendations about the need for improved monitoring, but there does not seem to be clear evidence from the results of this paper pointing to that conclusion. Similarly, conclusions presented about the issue of service providers in the area are conflicting. In the results, only 23% of participants reported a lack of service providers as a key limitation to treating FS, but increasing the amount of service providers in the area is presented as a key recommendation, which does not seem to be supported by the results. Conclusions and recommendations should only be based on the data and scope presented in this paper.

### Response

Thank you for this feedback. We agree that the original text that recommended improved monitoring of household behaviors was too strongly written and have modified the text to soften our recommendation while still highlighting the need to follow up on how households actually use SMOSS systems like ADPs. Second, to address your comment about a lack of service providers, we have re-written that paragraph to contextualize our recommendation and provide nuance that connects the study’s results to our recommendations. We hope that these changes satisfy your suggestions.

## Comment 14

Results and discussion should be separated into two distinct sections, with the results section first presenting the objective results of the analyses (preferably using tables and figures to succinctly summarize findings), and the discussion section interpreting the results.

### Response

Thank you for this feedback. While we understand your feedback and concerns, we have chosen to present the results and associated discussion topics within the same section (“Results and Discussion”) due to the complexity of the topics addressed in this article. We originally wrote the article with a separate Results and Discussion section but found that separating the results from the discussion produced an article that was more difficult to read and understand, particularly within the context of the existing literature. We checked if this was allowed by PLOS ONE before submitting, and it is allowed: as shown in [PLOS ONE submission guidelines](https://journals.plos.org/plosone/s/submission-guidelines), “The following elements can be renamed as needed and presented in any order: Materials and Methods, Results, Discussion, Conclusions (optional).” There are also many examples of PLOS ONE articles about sanitation that present results and discussion together; here are two: [Comparison of the efficiency of Deebag and jute made bag for faecal sludge management and wastewater treatment | PLOS One](https://journals.plos.org/plosone/article?id=10.1371/journal.pone.0241046) and [The SaniPath Exposure Assessment Tool: A quantitative approach for assessing exposure to fecal contamination through multiple pathways in low resource urban settlements | PLOS One](https://journals.plos.org/plosone/article?id=10.1371/journal.pone.0234364).

Regarding the second part of your feedback, we have already presented key findings succinctly in a summary table (Table 1) and use that table to frame the Results and Discussion section. If there are specific suggestions you have to improve this table, we would be happy to address those suggestions.

Based on our reasoning above, we have not made any changes to the article based on this feedback.

## Comment 15

It would be helpful to summarize findings from the companion article mentioned looking at microbial risks associated with ADPs. Additionally, details on the ADP management process were unclear and often confusing. The specific recommendations from iDE should be explained in detail in the introduction (not in a footnote), and it should be made more clear what procedures should be carried out by service providers and what should be done by households.

### Response

Thank you for this feedback. We have added a summary of the companion article to the Introduction and agree that it provides useful framing for the rest of this article. We have also added an expanded description of the recommended practices for ADP installation and operation to the text in the Introduction, as you suggested.

## Comment 16

It would be helpful to align the section headers in the methods and results sections to make it easier to follow the distinct research questions and analyses conducted.

### Response

Thank you for this feedback. Unfortunately, we are confused about what your comment means: the word “align” is unclear and could refer to a few different aspects of the manuscript, topics, or formatting. Could you provide more clarity about what you mean, particularly regarding “align”? We would be happy to address this comment upon clarification. Thank you for your understanding.
