# Supplementary material for: Household perceptions, practices, and experiences with real-world alternating dual-pit latrines treated with storage and lime in rural Cambodia
Source: PLoS One. 2025 Oct 17;20(10):e0332118. doi: 10.1371/journal.pone.0332118 (PMC12533883; doi:10.1371/journal.pone.0332118)
Supplement: Attachment — Submitted filename: Response_to_Reviewers_auresp_2.docx [file pone.0332118.s016.docx]

Response to Reviewer Comments

PONE-D-24-32665

Household Perceptions, Practices, and Experiences with Real-world Alternating Dual-Pit Latrines Treated with Storage and Lime in Rural Cambodia

James Harper, PE, PhD and co-authors

July 22, 2025

Thank you for your comments on this article. We have addressed and responded to all of your comments and believe that it is now ready for publishing with PLOS ONE. Please see below for details.

# REVIEWER 2

## Comment 1

This resubmission summarizes data collected during surveys of people using alternating dual-pit latrines (ADPs) across 5 rural provinces in Cambodia, a previously understudied safely managed sanitation system. The authors used descriptive summaries of the data to assess the population’s sanitation practices and knowledge and developed indices summarizing various aspects of sanitation use to assess factors influencing sanitation practices, knowledge, and values. The authors conclude that while ADPs have the potential to safely treat FS, low adherence to safe management practices by users may limit ADP’s ability be an effective technology for achieving safely managed sanitation in Cambodia.

With revisions to the manuscript, the authors appropriately addressed comments and improved the quality of the manuscript, particularly the description of the methods and interpretation of results. The authors provided important justification for model selection, including improved descriptions of variables and clarification about index weighting, that helps to validate their analysis approach. Additionally, the improved language in the discussion and results align more appropriately with the findings from the analyses.

### Response

Thank you for this comment. We are happy that you are pleased with our revisions.

## Comment 2

There is one minor revision that would help provide important context for the reader - The information provided in supplemental file 3 is a valuable addition to this paper and would be helpful to include in the main body of the paper to inform the reader about important characteristics of the population. In particular, the summary on sanitation sharing and previous sanitation ownership included in S3 would be helpful to include in the main text to give readers insight into sanitation experiences in the study population. One key concern that should be addressed is that Table S3.6 is referenced in the supplemental file but does not appear to be included.

### Response

Thank you for this comment. We agree and have incorporated a simplified version of the text in Supplementary File 3 into the main text. We have also chosen to retain Supplementary File 3 for completeness.
